# Comprehensive molecular profiling of 718 Multiple Myelomas reveals significant differences in mutation frequencies between African and European descent cases

**DOI:** 10.1371/journal.pgen.1007087

**Published:** 2017-11-22

**Authors:** Zarko Manojlovic, Austin Christofferson, Winnie S. Liang, Jessica Aldrich, Megan Washington, Shukmei Wong, Daniel Rohrer, Scott Jewell, Rick A. Kittles, Mary Derome, Daniel Auclair, David Wesley Craig, Jonathan Keats, John D. Carpten

**Affiliations:** 1 Department of Translational Genomics, Keck School of Medicine, University of Southern California, Los Angeles, CA, United States of America; 2 Translational Genomics Research Institute, Phoenix, AZ, United States of America; 3 Van Andel Research Institute, Grand Rapids, MI, United States of America; 4 Department of Surgery, Division of Population Genetics, University of Arizona, Tuscon, AZ, United States of America; 5 Multiple Myeloma Research Foundation, Norwalk, CT, United States of America; UNITED STATES

## Abstract

Multiple Myeloma (MM) is a plasma cell malignancy with significantly greater incidence and mortality rates among African Americans (AA) compared to Caucasians (CA). The overall goal of this study is to elucidate differences in molecular alterations in MM as a function of self-reported race and genetic ancestry. Our study utilized somatic whole exome, RNA-sequencing, and correlated clinical data from 718 MM patients from the Multiple Myeloma Research Foundation CoMMpass study Interim Analysis 9. Somatic mutational analyses based upon self-reported race corrected for ancestry revealed significant differences in mutation frequency between groups. Of interest, *BCL7A*, *BRWD3*, *and AUTS2* demonstrate significantly higher mutation frequencies among AA cases. These genes are all involved in translocations in B-cell malignancies. Moreover, we detected a significant difference in mutation frequency of *TP53* and *IRF4* with frequencies higher among CA cases. Our study provides rationale for interrogating diverse tumor cohorts to best understand tumor genomics across populations.

## Author summary

This study represents the largest comprehensive molecular analysis of ethnically defined newly diagnosed treatment naïve Multiple Myeloma (MM). We revealed significant differences in mutation frequencies for important cancer genes between AA and CA MM. This study provides support for interrogating diverse tumor cohorts to best understand tumor genomics across populations.

## Introduction

Multiple Myeloma (MM) is a malignancy of plasma cells provoked by immunoglobulin gene rearrangements, accounting for slightly more than 10% of all hematologic cancer diagnoses in the US [1–3]. Pathogenesis evolves from an asymptomatic premalignant stage of clonal plasma cell proliferation termed “monoclonal gammopathy of undetermined significance” (MGUS) [4, 5]. MGUS is present in more than 3% of the population above the age of 50 and progresses to MM, or related malignancy at a rate of 1% per year [2, 6]. High-levels of pathogenic heterogeneity in MM among distinct racial/ethnic distributions have been outlined by epidemiology [7]. For instance, African American (AA) patients matched for socioeconomics, age, and gender are three times more likely to be diagnosed with MM, and with death rates that double those observed among Caucasians (CA) [4, 8, 9]. In addition, reports have shown that AA have decreased frequency of IgM monoclonal gammopathy, and have an earlier age of onset compared to CA patients [4, 8, 10]. However, over the past decade there were effective improvements in treatments and disease management that contributed to an astonishing increase in overall survival for MM, but these improvements were observed predominantly in CA patients [10, 11]. Therefore, a deeper understanding of oncogenic processes driving MM pathogenesis in statistically powered multi-ethnic cohorts is still needed to addressing disparities in incidence and outcomes observed among AA or otherwise African descent patients.

Several previous profiling studies have provided a view of the somatic landscape of MM [12, 13]. However, the representation of tumors from AA has been critically limited. To date, only one study has been reported comparing the frequencies of molecular alterations in MM between AA and CA cases [14]. This study revealed lower frequencies of IgH translocations by Fluorescence In Situ Hybridization among AA [14]. Although seminal, the small sample size of tumors from AA, lack of coding mutation data, and incomplete access to clinical data represented limitations of this study [14].

Recently, a comprehensive longitudinal study (CoMMpass) was initiated with the overall goal to prospectively observe the natural history of MM through comprehensively profiling 1,000 MM cases at diagnosis, with multiple biological and clinical follow-up points throughout the course of clinical management. Genomic profiling includes whole exome sequencing of germline and MM tumors, low pass whole genome sequencing, and RNA sequencing of tumors. Data are publicly distributed as Interim Analyses throughout the course of the study. Interim Analysis 9 (IA9) includes whole exome sequencing (WES) data from bone marrow tumor extracts with matching normal from 796 newly diagnosed MM cases. Patient ethnicity was one of the demographic parameters collected for each patient that included self-identification categories for African American, Caucasian, Asian, Hispanic, Middle Eastern, Other, Declined, and Unknown. This enabled us to perform genomic analyses to assess potential somatic differences from tumors based upon self-reported race. Alternatively, genetic ancestry is characterized by population genetic informative markers derived from allele frequencies of single nucleotide variances across biogeographical distributions [15] and is another way to characterize populations and individuals. The strength of genetic ancestry is that it offers information on ancestral genetic contributions based upon percent admixture within a given individual. While genetic ancestry provides molecular information with direct biological implications within the context of a disease [16, 17], we cannot disregard the importance of self-reporting that is influenced by socio-environmental behaviors that also play a critical role in disease risk. Most MM studies to date have been based upon self-reported race information. However, the availability of self-reported race and WES data from CoMMpass IA9 provided the unprecedented opportunity to search for novel and statistically significant somatic alterations relative to ancestrally-defined population differences in MM cases.

## Results

### CoMMpass IA9 data metrics

For this study, we utilized a subset of the CoMMpass IA9 dataset comprised of high quality WES and RNA-seq data from 721 newly diagnosed MM patients that were self-reported as either AA (N = 128) or CA (N = 593) with longitudinal clinical follow-up (S1 Table). For the CoMMpass study, MM tumor specimens were enriched from bone marrow aspirates by CD138 antibody conjugation yielding on average 99% CD138+ plasma tumor cell purity. Genomic data utilized for the downstream analysis for this study includes matching germline-tumor WES data on 718 MM cases (S1 Table). Sequencing statistics are provided in (S2 Table). For WES data, we achieved a mean coverage of 124X for MM tumor samples and 126X for germline samples. The average percentage of reads mapping to the WES target regions at 30X was 93% for tumor samples and 94% for germline samples. For RNA-seq, we generated an average of 200 million read pairs per sample with 88% mapping on average to annotated gene regions.

### Characterization of CoMMpass IA9 cases by genetic ancestry

**Fig 1:**
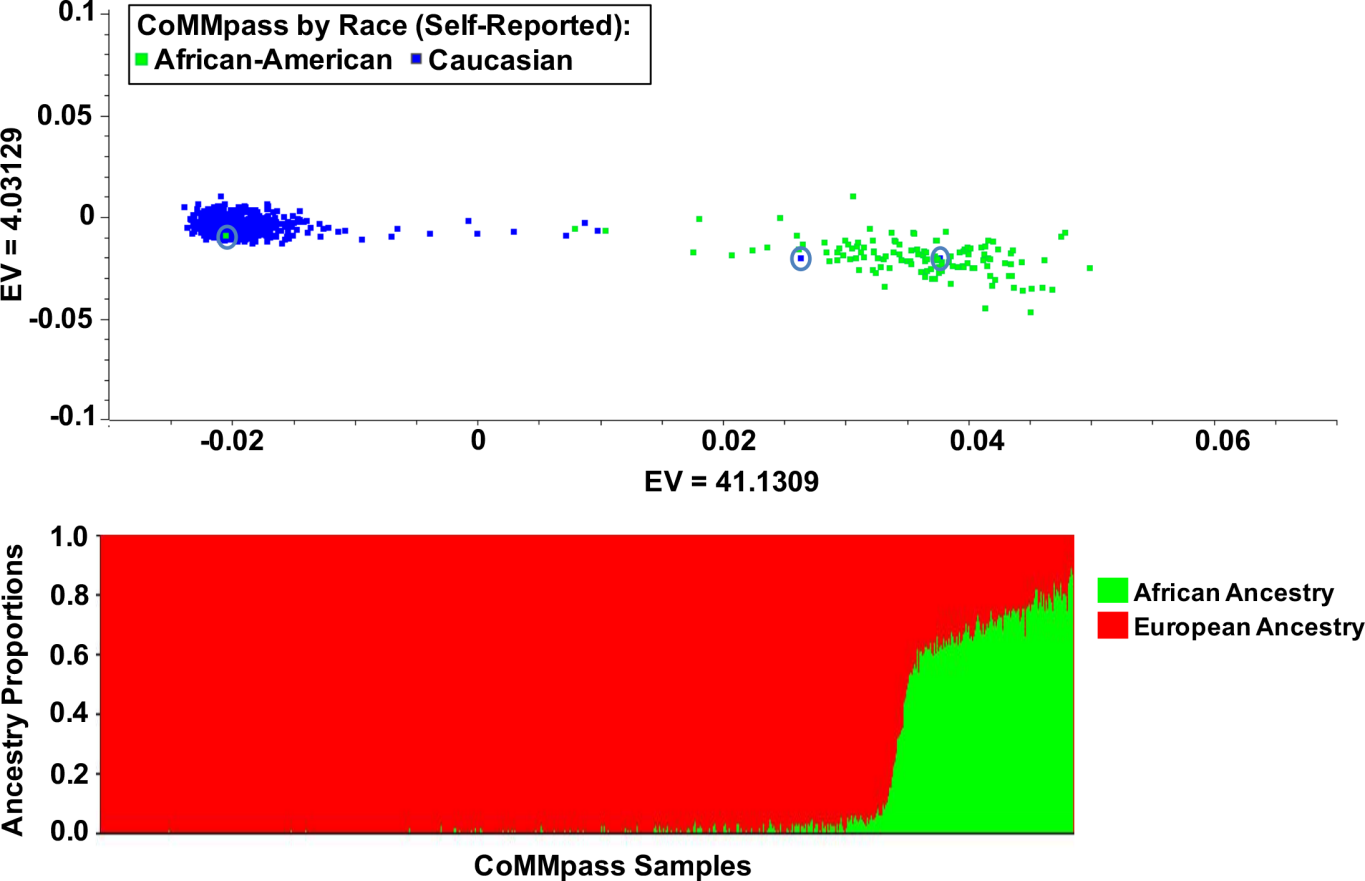
Population stratification by principal component analysis and STRUCTURE plot defining propositions of calculated genetic ancestry. (A) Principal component plot across all samples by self-reported race with Caucasian (blue dots) and African American (green dots) using AIMs derived from the whole-exome deep sequencing. The red circles indicate samples that have been removed from the analysis due to misclustering. PCA is calculated using SNP & Variation Suite v8.4.1 (Golden Helix, Inc.) PCA tool by eigenvalue (EV) implementation technique (Methadology). These samples were identified as the self-reported race. (B) STRUCTURE plot (K = 2; 50,000 Burnin period and 100,000 MCMC repeats) used to interfere genetic clusters and percent admixture: European Ancestry (red), African Ancestry (green).

To delineate genetic ancestry, we used a population stratification principal component analysis (PCA) to cluster MM patients by extracting 4,761 Ancestry Informative Markers (AIMs) SNP genotypes derived from germline WES CoMMpass IA9 data (Fig 1A). This allowed us to assess the distribution of genetic ancestry for CoMMpass cases [18]. We also utilized STRUCTURE [19] to determine individual percent ancestry for each CoMMpass case (Fig 1B, S1 Table). Analysis of individual ancestry data revealed that two self-reported CA had greater than 55% African ancestry and one self-reported African American had 99.9% European ancestry (Fig 1A, red circles, S1 Table). These three cases were excluded from our analyses. This resulted in a total of 127 African American and 591 Caucasians that were used for all downstream analyses. The mean European admixture among self-reported AA was 31% (range; 11%–67.8%). The mean west African admixture among self-reported CA was 0.1% (range; 0–34.3).

**Fig 2:**
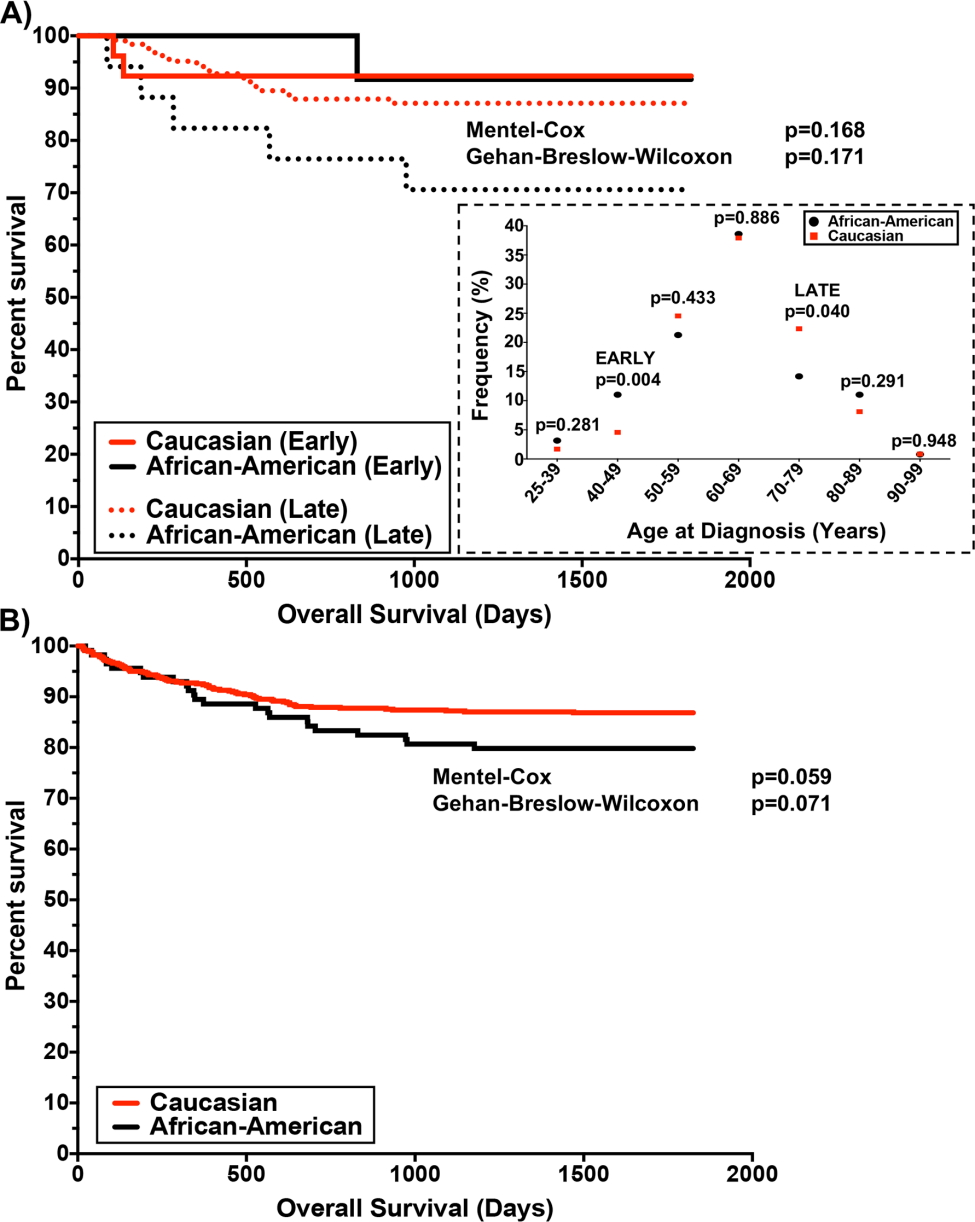
Overall survival of patients based upon *age of MM onset and race*. (A) Kaplan-Meier analysis with long-rank test for overall survival data from CoMMpass IA9 cases stratified by the impact of the early or late onset of MM. The data in the black box demonstrate the distribution of onset across the IA9 data set. The early (term used to label onset between 40–49 years), and late (term used to label onset between 70–79 years) that were significantly different using Fisher’s exact test between the stratified populations. Samples size also summarized in supplemental table (S3 Table) is as following: 25–39 [AA (4/127) vs CA (10/591)], 40–49 [AA (14/127) vs CA (27/591)], 50–59 [AA (27/127) vs CA (145/591)], 60–69 [AA (49/127) vs CA (224/591)], 70–79 [AA (118/127) vs CA (132/591)], 80–89 [AA (14/127) vs CA (48/591)], 90–99 [AA (1/127) vs CA (5/591)]. (B) Kaplan-Meier analysis with long-rank test for overall survival data from CoMMpass IA9 impact of incidents of MM by race-matched-ancestry.

Analysis of demographics data stratified by race confirmed the previously reported finding by Waxman et al. [10] of a significant (p = 0.004; Fisher’s) two-fold increase in early age of onset (40–49 years) of MM among AA cases (11%) compared to CA cases (4.6%) (Fig 2A, S3 Table). In addition, there was a reverse effect in later ages of onset (70–79 years) with significantly higher frequency (p = 0.04; Fisher’s) in CA (22%) compared to AA (14%) (Fig 2A, S3 Table). Interestingly, our data showed no significant difference in overall survival based upon, race, age of onset, and MM karyotype in this cohort of similarly treated MM cases (Fig 2A and 2B, S1 Fig).

### Comparison of somatic mutation profiles between AA and CA MM cases

**Fig 3:**
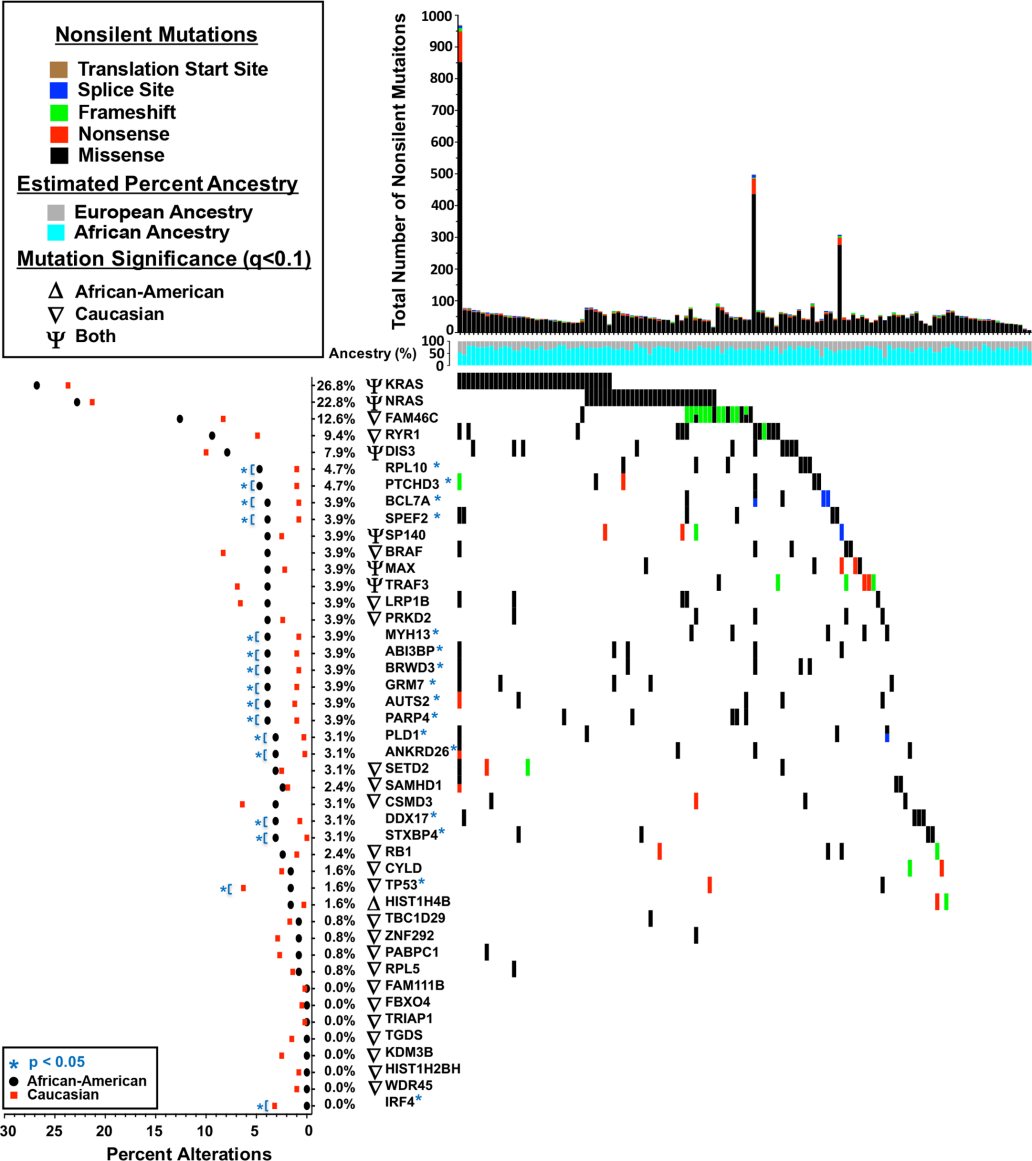
Frequencies of recurrently mutated genes in human MM. (Top) The total number of acquired nonsilent somatic mutations across 127 tumors from African American patients. Percent ancestry track is indicating the distribution of genetic ancestry among each sample. (Center) mutations across recurrent genes among African American patients colored by mutation type. (Left) Mutation significance analysis was performed using MutSigCV (Methods) on two cohorts African American (n = 127) and Caucasian (n = 591) independently. For each analysis, significance was determined using false discovery rate (q<0.1) with significant genes labeled as following: exclusive to African American (Δ) and Caucasian (∇), or if the same gene is identified by both analysis “Both” (ѱ). (Left) Histogram depicting percent of alterations in each gene between African American (black) and Caucasian (red) cohorts using the Fisher’s exact test with significance set at (blue; * = p<0.05) between the two stratified populations.

Somatic mutational analysis of the WES data was performed using a modified Mutation Significance (MutSig CV) algorithm [20] with custom scripts designed to detect differentially mutated genes between AA and CA MM tumors (Fig 3). We did not detect a statistically significant difference in mean nonsynonymous mutation frequencies between AA (mean = 63) and CA (mean = 68) MM cases (p = 0.574) (S3A Fig). Furthermore, there was no difference in the mutational signature between AA and CA MM cases (S3B Fig). Somatic mutational analysis across the entire cohort confirmed common mutated MM driver genes such as *KRAS*, *NRAS*, *BRAF*, *TP53*, *DIS3*, *and FAM46C* (Fig 3, S4 Table) [12, 13, 21]. Our comparison of mutated genes between tumors from AA and CA cases identified *RYR1*, *RPL10*, *PTCHD3*, *BCL7A*, *SPEF2*, *MYH13*, *ABI3BP*, *BRWD3*, *GRM7*, *AUTS2*, *PARP4*, *PLD1*, *ANKRD26*, *DDX17 and STXBP4* as genes with significantly higher mutation frequencies in AA MM cases (Fig 3, S4 Table). *FAM46C*, which is commonly mutated in MM, also exhibited a trend toward higher frequency in AA (12.6%) versus CA (8.3%) MM, however the difference did not reach statistical significance (p = 0.09). In addition, we further examined differences in BRAF mutation frequency between AA and CA cases. Although we did not detect a difference in overall BRAF mutation frequency, we did observe a difference in BRAF^V600E^ mutation between AA (0.8%) versus CA (4.34%), but this difference did not reach nonimnal significance (p = 0.053; Fisher’s) (S1 Table).

**Fig 4:**
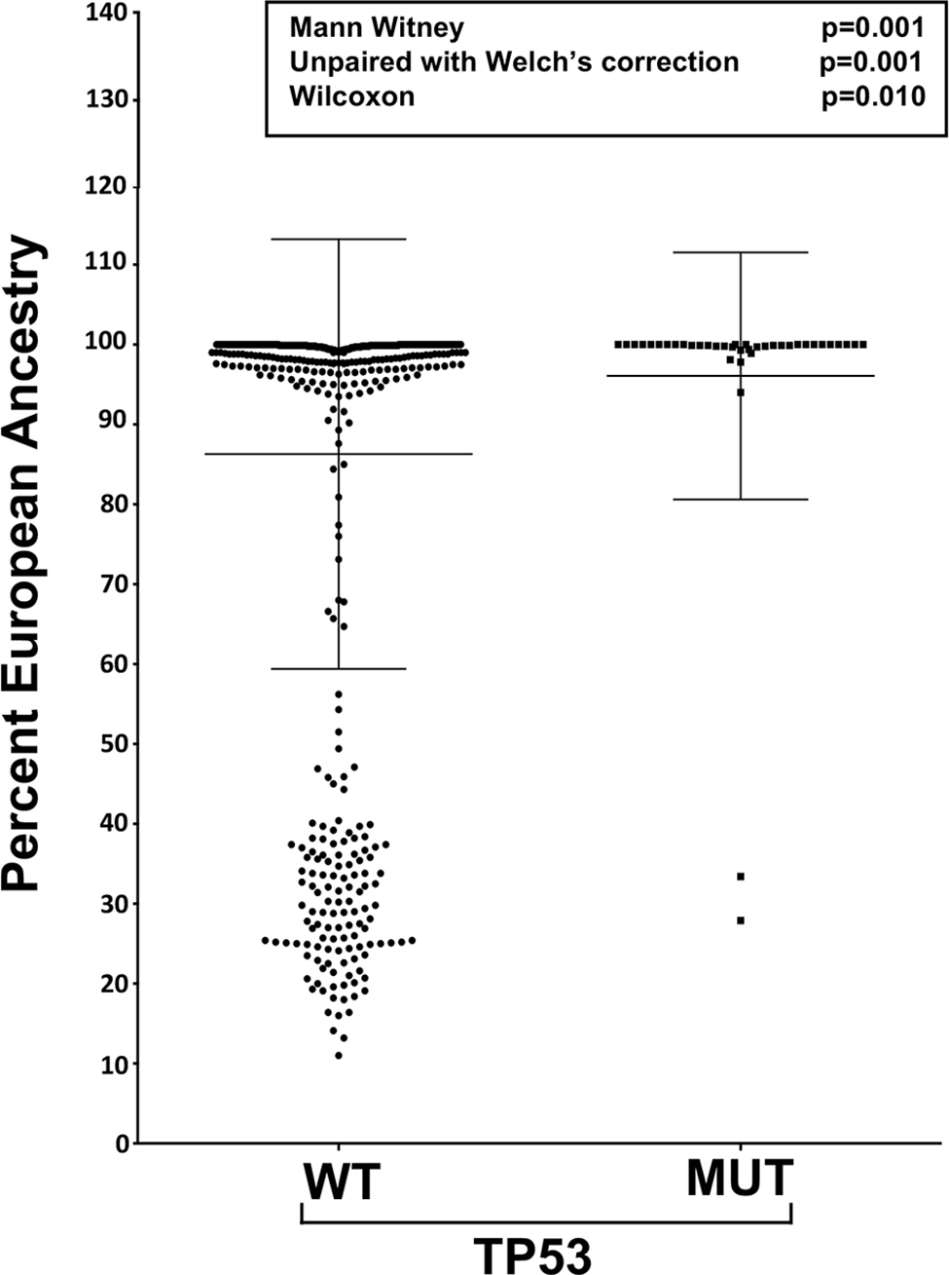
Alteration of TP-53 across ancestry. Analysis of TP53 state across the percent European ancestry. Each dot represents individual sample. To validate statistical power, we performed Mann-Witney, Wilcoxon, and Unpaired t-test with Welch’s correction with statistical significance set at p<0.05.

Among the most interesting observations of our mutational analysis was the significantly higher frequency of *IRF4* (p = 0.041) and *TP53* (p = 0.035) mutations in CA MM cases (Fig 3, S4 Table). Specifically, this analysis revealed a *TP53* somatic mutation frequency of 6.3% in the CA MM cases compared to 1.6% in AA MM cases (p = 0.035) (Fig 3, Table 1, S2 Fig, S4 Table). To verify our observation, we used an independent publically available MM somatic whole exome sequencing dataset consisting of 205 MM cases as a validation cohort published by Lohr et al (S5 Table) [13]. This dataset consisted of a mix of newly diagnosed and relapse MM specimens. Although there were only 14 self-reported AA MM cases within this validation cohort, we observed differences in *TP53* coding mutations compared between CA (14/157; 8.9%) and AA (0/14; 0%), providing an independent validation, albeit with limited power. Given the significantly higher *TP53* mutation rate among CA MM cases prompted us to assess the distribution of *TP53* mutation status as a function of European ancestry. The analysis demonstrated that *TP53* mutations were strongly associated with MM cases that have high European ancestry (>95%) (p = 0.01; Wilcoxon rank-sum test) (Fig 4).

**Table 1:** TP53 mutation profile.

TP53 Aberration	African-American	Caucasian	p-value (Fisher's)
Wildtype	84.3%	83.1%	0.75
Mono-Alleic	9.4%	9.1%	0.91
Bi-Alleic	0.8%	4.1%	0.07
LOH	6.3%	8.6%	0.39
Mutation	1.6%	6.6%	0.03
LOH+Mutation	7.9%	15.2%	0.03

Wildtype (both TP53 alleles are normal); Mono-Allelic (somatic mutation or copy loss event); Bi-Allelic (somatic TP53 event in both alleles); LOH (loss of heterozygosity only), Mutation (somatic mutation only); LOH+Mutation (combined loss of heterozygosity and mutations)

### Comparison of somatic copy number changes between AA and CA MM cases

**Fig 5:**
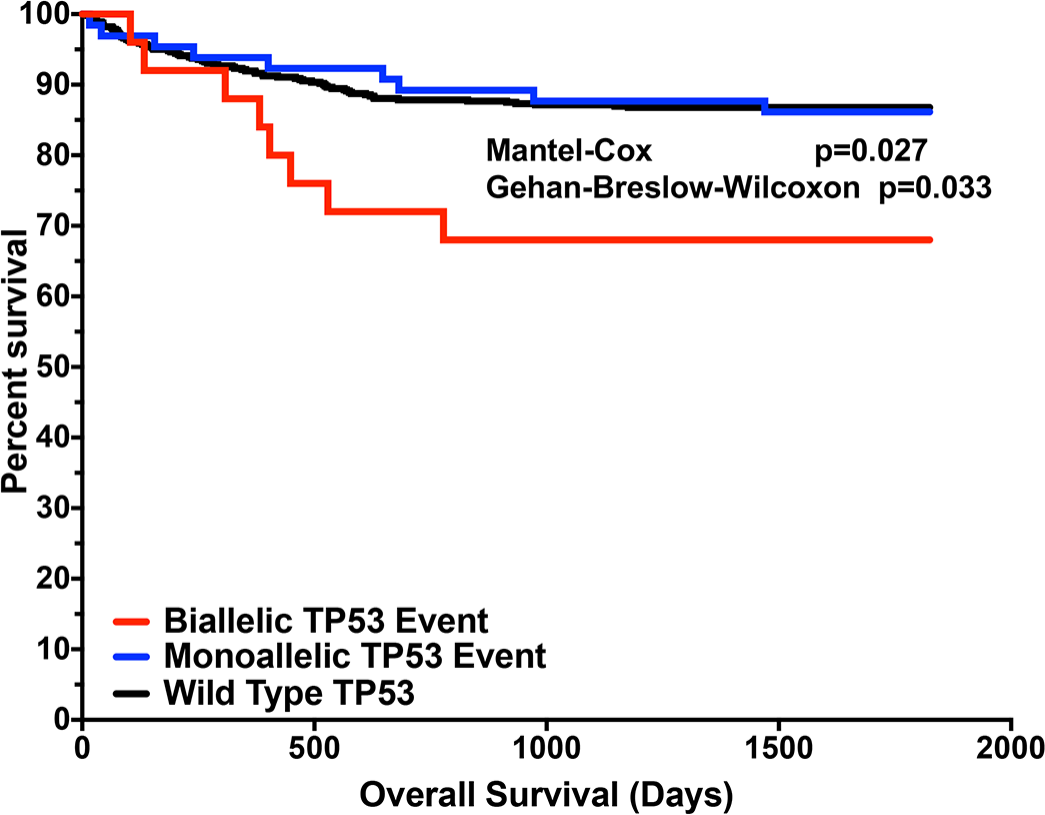
Overall survival of patients based upon *TP53* locus alterations. (A) Kaplan-Meier analysis with long-rank test for overall survival data from CoMMpass IA9 cases stratified by mono-allelic (blue) versus bi-allelic (red) alteration as well as wildtype (black) of the TP53 locus.

To uncover potential racial or ancestry differences in copy number events across CoMMpass IA9 WES data, we utilized GISTIC 2.0 analysis [20, 22]. This analysis identified several regions of the genome associated with common copy number gain and loss (S4 Fig, S6 Table). However, we did not detect any statistically significant differences in specific focal copy number events between AA and CA cases. However, to further expand upon our understanding of *TP53* loss based upon mutational analysis data, an integrated somatic copy number and mutational analysis of the *TP53* locus was performed. This analysis revealed a predominance of bi-allelic *TP53* events among CA MM cases (Table 1), however the difference was not statistically significant. Furthermore, integration of genomic data with clinical outcomes demonstrated that CoMMpass cases with tumors harboring bi-allelic perturbation in *TP53* have significantly (p = 0.027; Mental-Cox Log-rank test) poorer overall survival (Fig 5). However, there is no difference in overall survival in MM patients with tumors demonstrating mono-allelic events (loss of copy, or mutation only) and wild type *TP53* (Fig 5).

### Assessment of MM transcriptional signatures among MM derived from AA and CA cases

We performed gene expression profile analysis of RNA-seq data to compare the frequency of the University of Arkansas UAMC 70 gene high-risk signature between AA and CA MM cases [23]. Comparison between African and European ancestry and by self-reported race further sub-stratified by MM karyotype did not reveal a significant difference (p = 0.662) of the UAMC high-risk signature consistent with our previous report from an independent data set (Table 2) [14]. Further analysis of Ki67 proliferation index showed no significant difference (p = 0.560) of Ki67 profile among patients stratified by race or ancestry (S5 Fig)

**Table 2:** Frequency analysis of Arkansas 70 high-risk genes expression profile.

Risk Groups	Frequency AA (%)	Frequency E A (%)	p value (Fisher’s Exact)
**Total**	28	26	0.662
**Hyperdiploid**	20	20	0.999
**Nonhyperdiploid**	33	33	0.935

## Discussion

Not surprisingly, the vast majority of our current understanding of MM biology has been derived from data collected from largely European descent cohorts, even given significant disparities in disease incidence and outcomes seen among African American patients [10]. Through the analysis of CoMMpass data, we were able to analyze the largest multi-ethnic MM cohort at diagnosis to date, in an attempt to elucidate in-depth molecular differences in tumors derived from AA and CA cases to further understand the biological determinants of MM as a function of tumors derived from an ancestrally defined dataset. First, the nature of the CoMMpass dataset has allowed us to confirm previously reported results of published clinical and molecular studies of MM. Our study results were similar to previously reported data demonstrating earlier disease onset in MM among African Americans [10]. Furthermore, several MM genomics sequencing studies [12, 24, 25] have been reported in both newly diagnosed and relapse MM and we have validated commonly mutated genes in MM such as *KRAS*, *NRAS*, *FAM46C*, *DIS3 and TP53*.

Differences in mutation frequencies in genes such as *RYR1*, *RPL10*, *PTCHD3*, *BCL7A*, *SPEF2*, *MYH13*, *ABI3BP*, *BRWD3*, *GRM7*, *AUTS2*, *PARP4*, *PLD1*, *ANKRD26*, *DDX17 and STXBP4* that were more common in AA MM cases could possibly reflect differences of myelomagenesis by race and/or ancestry. *FAM46C* is among the most commonly mutated genes in MM based on several previously reported WES studies [12]. The reported *FAM46C* mutation frequency in MM ranges from 5%-11% [13, 26], and although the frequency was 8.3% in Caucasian cases in our study, we observed an increased frequency of 12.6% in African American cases in our study. Although the functional role of *FAM46C* in myelomagenesis is still yet to be determined, there seems to be a potential enrichment of its role in MM biology among tumors derived from patients of African ancestry.

BRAF mutations have also been reported in MM, generally at frequencies of 2.8%-5% [27–29]. Although overall BRAF mutation frequencies were not different between AA and CA, we did detect differences when stratifying specifically by BRAF^V600E^, with higher frequencies seen in CA (4%) as compared to AA (0.8%), although this differenece did not exceed nominal significance due to limited power. Furthermore, we did not observe a difference in overall survival in primary MM cases harboring any BRAF mutation, nor specifically BRAF^V600E^ mutation compared to BRAF wildtype cases (p = 0.439, p = 0.579; Long-rank, Gehan-Breslow-Wilcoxon). These results are of clinical consequence as BRAF mutations, particularly BRAF^V600E^, can be targeted with select BRAF inhibitors, which has shown effect in mutation positive MM cases [27, 29].

Three of the genes with significantly higher mutation frequency in AA are involved in other B-cell malignancies. *BCL7A* has been shown to be directly involved in a three-way gene translocation with Myc and IgH in high-grade B-cell non-Hodgkin lymphoma cell lines [30]. As a result of the gene translocation, the N-terminal region of the gene product is disrupted, which is thought to be related to the pathogenesis of a subset of high-grade B cell non-Hodgkin lymphoma [30]. The protein encoded by *BRWD3* is a bromodomain and WD repeat containing protein that is thought to have chromatin-modifying function, and may play a role JAK/STAT pathway activity [31]. Importantly, this gene is involved in translocations in B-cell chronic lymphocytic leukemia [32]. Finally, *AUTS2* is involved in translocations with PAX5 in B-cell precursor acute lymphoblastic leukemia and other cancers [33]. These findings are of considerable interest, and further validation and functional characterization of these genes in appropriate myeloma model systems could shed light on their potential role in myelomagenesis particularly in patients of African descent.

Another significant observation of our study was the difference in *TP53* mutation frequency among patients with higher European admixture in our dataset. Integrated mutation and copy number analysis demonstrated a trend towards higher *TP53* bi-allelic inactivation in tumors derived from CA cases. This could have translational significance as bi-allelic *TP53* inactivation is a universally validated predictor of poor outcome [34]. We further detected significant differences in *IRF4* mutation frequency, which was also higher among CA MM cases. *IRF4* is a credentialed MM oncogene, which is known to be an oncogenic fusion partner in MM, and is also among the known significantly mutated genes in MM [35, 36]. Moreover, IRF4 activity is associated with poor outcome, with potential therapeutic implications for immunomodulatory agents in MM [37, 38]. These data suggest that the significant enrichment of *IRF4* mutations in CA cases could have strong clinical translational implications. Although the collection of longitudinal clinical data is ongoing for CoMMpass, one current limitation of this study is the limited long-term follow-up data from IA9. However, the CoMMpass study design will allow us to assess outcomes longitudinally, along with molecular profiles of both newly diagnosed and relapse disease in upwards of 1,000 MM cases.

Ultimately, our study represents among the most comprehensive (WES and RNAseq) genomics studies of a tumor type in patients of African descent, and sheds light on potential ancestry-related differences in biological mechanisms of myelomagenesis. Three genes that are known to be involved in B-cell malignancy translocations represent new candidate myeloma genes that may have been overlooked because of the lack of AA cases in most large genomic efforts. It is clear that there are molecular differences between MM tumors from AA and CA cases, and that it is absolutely critical to continue to delineate these observations to better improve clinical management of the disease. As the CoMMpass study matures, it will allow scientists to validate these findings as well as expand on studies such as recurrence, better elucidation of driving mutations, and clonal evolution during the course of treatment.

## Materials and methods

### Ethics statement

Samples were obtained under Multiple Myeloma Research Foundation (MMRF) CoMMpass Study Network Institutional Review Board approved Informed Consent; Copernicus IRB (IRB # QUI1-11-217).

### CoMMPass study synopsis

The design of the study is to prospectively profile newly diagnosed, treatment naive MM from 1000 patients with longitudinal clinical follow up. Tumor specimens collected at diagnosis and relapse are interrogated by whole-exome, modified low pass whole-genome, and or RNA sequencing. The longitudinal component requires clinical follow-up of each patient with collection of clinical parameters four times annually over a period of 10 years. Furthermore, each patient participating into the study underwent an IMID and/or Proteasome inhibitor based treatment regimen at diagnosis determined by the treating oncologist.

CoMMpass data are systematically analyzed and periodically released in the form of Interim Analyses on a biannual basis. Interim Analysis 9 (IA9) is comprised of 796 unique baseline newly diagnosed bone-marrow samples with high quality WES data of which, 75 have confirmed progression with comprehensive clinical annotation. In addition, IA9 is comprised of 520 bone marrow baseline samples that were analyzed by both whole-exome and RNAseq platforms. The data is publically available at dbGAP accession number phs000748.

In this study, we performed our analysis on whole-exome data from 741 of treatment-naïve bone marrow derived MM matched normal samples from IA9, from those cases who self-reported race as either African American or Caucasian. To ensure high quality WES data for downstream copy number analysis, we removed samples that had maximum segmentation count above 2,500 to be in concordance with GISTIC 2.0 recommendation of maximum segment counts [22]. This resulted in final 721 samples that passed the quality threshold and were used to determine genetic ancestry across the samples.

### Sample preparation

Bone marrow aspirates and peripheral blood samples were collected from each patient. Bone marrow aspirates from each patient were subjected to immunomagnetic bead separation using the Miltenyi MACS Cell Separation System (Miltenyi, San Diego, CA) to enrich for CD138-positive malignant MM plasma cells. Only clinically eligible samples with greater than 250,000 cells recovered after CD138 enrichment, which are greater than 80% monoclonal light chain restricted plasma cells move forward for nucleic acid extraction. Genomic DNA was extracted from purified CD138-positive plasma cells (tumor) and matched peripheral blood samples (constitutional) using QIAamp DNA Mini Kit (Qiagen). Total RNA was extracted from CD138-positive plasma cells the using QiaAmp RNeasy Mini Kit (Qiagen). Nucleic acids were quality assessed using the Qubit 2.0 (Thermo Fisher) and Agilent Tape Station to determine quantity and integrity. Samples were stored at -80°C for subsequent molecular analyses.

### Massively parallel sequencing of DNA and RNA from CoMMpass specimens

DNA samples were used for two different assays including whole exome sequencing (WES) and low pass long insert whole genome sequencing (WGS). WES was prioritized if material was limiting. For WES, 50ng-250ng of genomic DNA was fragmented to an average size of 180bp in length using a Covaris focused-ultrasonicator (Covaris). An Illumina sequencing technology compatible whole genome library was created using Kapa Biosystems Hyper Prep Kits. These libraries were then subjected to whole exome target enrichment using Agilent SureSelect V5+UTR hybrid capture kits.

For RNA-sequencing, either 150ng or 500ng of total RNA was used to enrich for poly-adenylated RNA molecules, which were subsequently fragmented to a target size of 180bp by heat fragmentation. Fragmented molecules were then converted to cDNA using random primers with Superscript II (Invitrogen). After second strand synthesis, the resulting molecules were used for library prep using the Illumina TruSeqRNA library kit.

### Massively parallel sequencing

Parallel sequencing of libraries was performed on Illumina HiSeq2000 or HiSeq2500 systems using version 3 or version 4 chemistry. WES was sequenced using paired-end 83x83bp reads while long-insert whole genome libraries were sequenced using paired-end 86x86bp reads. All sequencing reads were converted to industry standard FASTQ files using BCL2FASTQ v1.8.4.

### Bioinformatics analysis of massively parallel sequencing data alignments

FASTQ files are processed using a custom semi-automated pipeline based upon industry standard software packages and programs. Sequencing reads are initially aligned to the GRCh37 human genome reference using v0.7.8 BWA-MEM aligner [39] to generate BAM files. SAMTOOLS v0.1.19 [40] was used to sort BAM files and PICARD v1.111 (http://broadinstitute.github.io/picard/) to mark duplicate read pairs. Post alignment joint INDEL realignment and base quality scores recalibration was performed on the BAM files using GATK v3.1–1 [41].

### Variant detection and mutational analysis

For WES data, final BAM files for each patient’s constitutional and tumor data were used for germline and somatic variant detection, respectively. Variants were called from germline and tumor BAM files individually using GATK Haplotype Caller v3.1–1 [42] and SAMTOOLS v0.1.19 [43]. Somatic mutations including single nucleotide variants SNVs and INDELs were called using each patients germline and tumor BAMs by three independent software packages including MUTECT [44], STRELKA [45], and SEURAT [46]. To make the final mutation list, a mutation had to be detected by at least two out of three independent callers used.

Somatic copy number analysis was performed on WES germline-tumor pairs. For these studies, we utilized the DNAcopy segmentation module in BioConductor [47]. We also utilized a comparative germline-tumor copy number approach where by raw data was normalized to physical coverage using circular binary segmentation as well as filtered to remove repetitive regions prior to calculating log2 comparison across germline-tumor exome data.

Somatic events were assembled in VCF and MAF formats and further annotated using SNPeff [48] to provide additional information on gene states and variant effects.

For RNA-sequencing analysis, we employed the TopHat v2.0.11 for alignment of RNA-seq reads, CuffDiff v2.2.1 for differential expression analysis, and Salmon 0.7.2 for isoform quantification [49].

### Secondary analysis of somatic alterations

Genotypes for germline variants with >98% detection across all samples with exome data, and used for somatic analysis, were deduced using the SNP & Variation Suite v8.4.1 (Golden Helix, Inc., Bozeman, MT, www.goldenhelix.com) genotype tool. Exome specific genome-wide Ancestry Informative Markers (AIMs) were derived from Kosoy et al. [50], Price et al. [51], Tandon et al. [52], and using informativeness estimation established by Rosenber et al. [53]. Population stratification principal component analysis (PCA) was calculated using the SNP & Variation Suite v8.4.1 (Golden Helix, Inc., Bozeman, MT, www.goldenhelix.com) Genotype PCA tool that implements eigenvalue technique described by Patterson et al. 2006 [54] and Price et al. 2006 [51]. The genotype file containing AIMs was further formatted for STRUCTURE analysis using plink and PGDSpider v2.1.0.3 [55]. STRUCTURE was performed as described by Pritchard et al. [19] with set Burning period for each replicate at 50,000 with consecutive 100,000 iterations of MCMC repetitions. Each genetic cluster was run with 3 independent replicates and the number of populations (K) was estimated by implementing both L(K) and ΔK [19]. The reference populations used for the putative ancestral populations were derived from publically available 1000G Population Exome Phase1_v3 Genotypes [18].

The analysis to identify significant driver mutations from WES somatic mutation data was performed using MUTSIG CV (Mutation Significance) algorithm [22] with adjusted covariates file using myeloma specific expression profile with significance set at q<0.1 and p<0.05. GISTIC 2.0 (The Genomic Identification of Significant Targets in Cancer) was applied to define significantly altered somatic copy number focal events with q value cut off set at 0.25 [20, 22]. Mutation signatures were deduced using an industry standard publically available analysis tool at https://bitbucket.org “analysis-of-mutational-signatures”.

### Genetic MM subgroups

Myeloma samples were further stratified by two subtypes: hyperdiploid or nonhyperdiploid. Hyperdeploidy was defined by presence of trisomy of at least three odd-numbered chromosomes [14]. The rest of the samples were identified as nonhyperdiploid subtype.

### High-Risk gene-expression signature

Gene expression profile was performed on RNA extracted from CD138+ plasma cells as described above using HiSeq2500 sequencer (Illumina, Inc.). RNA-seq Sailfish TPM values were log2 transformed prior to the analysis. To determine the high-risk score, we utilized the UAMC 70-gene expression profile [23]. The high-risk expression signature was calculated and reported as percent frequency across each ancestry and MM subtype.

### Mutation signature

Mutation signature was deduced using all somatic point mutations except INDELs using a publically available tool (https://bitbucket.org/jtr4v/analysis-of-mutational-signatures).

### Statistical analysis

Each event between the stratified populations was analyzed appropriately either using the Fisher’s exact test [56], non-parametric Mann-Whitney-Wilcoxon when assumption of normality is not maintained, and unpaired t-test with Welch’s correction when normal distribution is assumed with unequal variances. Benjamin-Hochberg method [57] was used to adjust for multiple testing. Overall survival was inferred from the clinical follow-up data collection over the 4 years’ spam with estimations using Kaplan-Meier methods. The p-value of < 0.05 was set for statistical significance. Statistical software packages used throughout the study were R v3.1.1. (https://www.r-project.org) and GraphPad Prism 7 (GraphPad Software, Inc.).

## Supporting information

S1 Fig Overall survival by multiple myeloma karyotypes. Analysis was performed using Kaplan-Meier method with long-rank test for group comparisons.
(TIF)

S2 Fig Mutation distribution across TP53 protein structure. cBioPortal Mutation Mapper tool as described by Gao et al. *Sci*. *Signal*. 2013 & Cerami et al. *Cancer Discov*. 2012 was applied to generate the mutation profile across the TP53 domains with top representing mutation profile among Caucasian, and bottom representing African American respectively.
(TIF)

S3 Fig Somatic mutation frequency of significantly mutated genes in tumors from African-American and Caucasian. (A) Comparison of nonsilent mutation frequency between ancestry and self-reporting using Wilcoxon, Mann-Whitney, and Unpaired t-test to determine statistical significance. (B) Mutation signature associated with African and European ancestry.
(TIF)

S4 Fig GISTIC 2.0 plot. Differentially altered events indicated by arrows. The red graph indicate copy number gains and blue is deletions for each stratified group.
(TIF)

S5 Fig Expression profile. Ki67 expression profile in TPM across patients with MM.
(TIF)

S1 Table Genomic data utilized, gender, reported race, and inferred genetic ancestry utilized in this study. (XLSX)

S2 Table Sequencing statistics matrix for Whole exome Sequencing (WES), and RNA sequencing. (RNAseq) used in this study.
(XLSX)

S3 Table Clinical outline, treatment summary, and karytype summary. (PDF)

S4 Table Summary of significantly mutated genes. (PDF)

S5 Table Data downloaded from Lohr, et al., cancer cell, 2014. 25(1).
(XLSX)

S6 Table Significant focal copy number perturbations called by GISTIC 2.0. (XLSX)
