# Supplementary figures and images for: Comprehensive molecular profiling of 718 Multiple Myelomas reveals significant differences in mutation frequencies between African and European descent cases
Source: PLoS Genet. 2017 Nov 22;13(11):e1007087. doi: 10.1371/journal.pgen.1007087 (PMC5699827; doi:10.1371/journal.pgen.1007087)

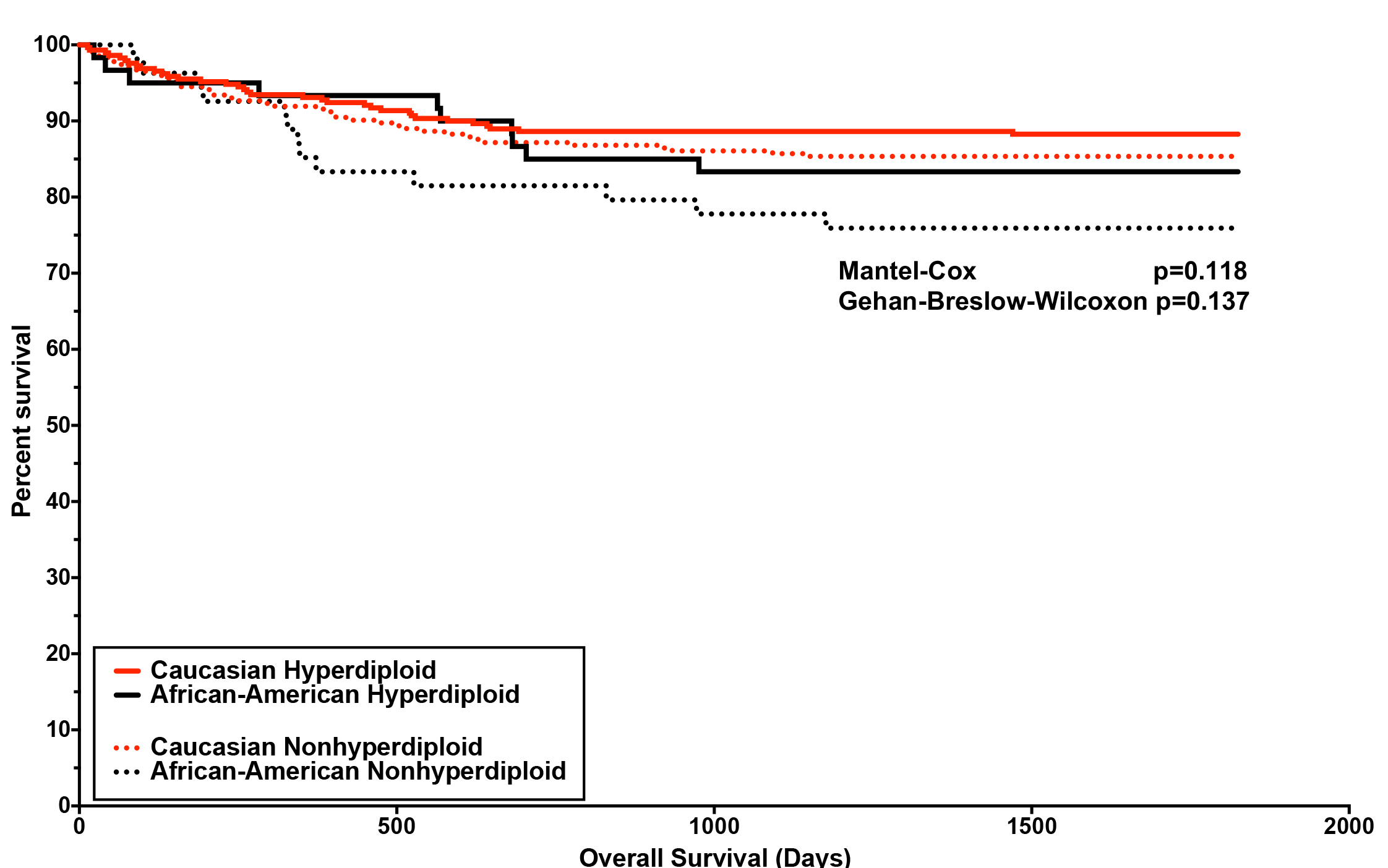

Supplement: S1 Fig — Analysis was performed using Kaplan-Meier method with long-rank test for group comparisons. (TIF) [file pgen.1007087.s001.tif]

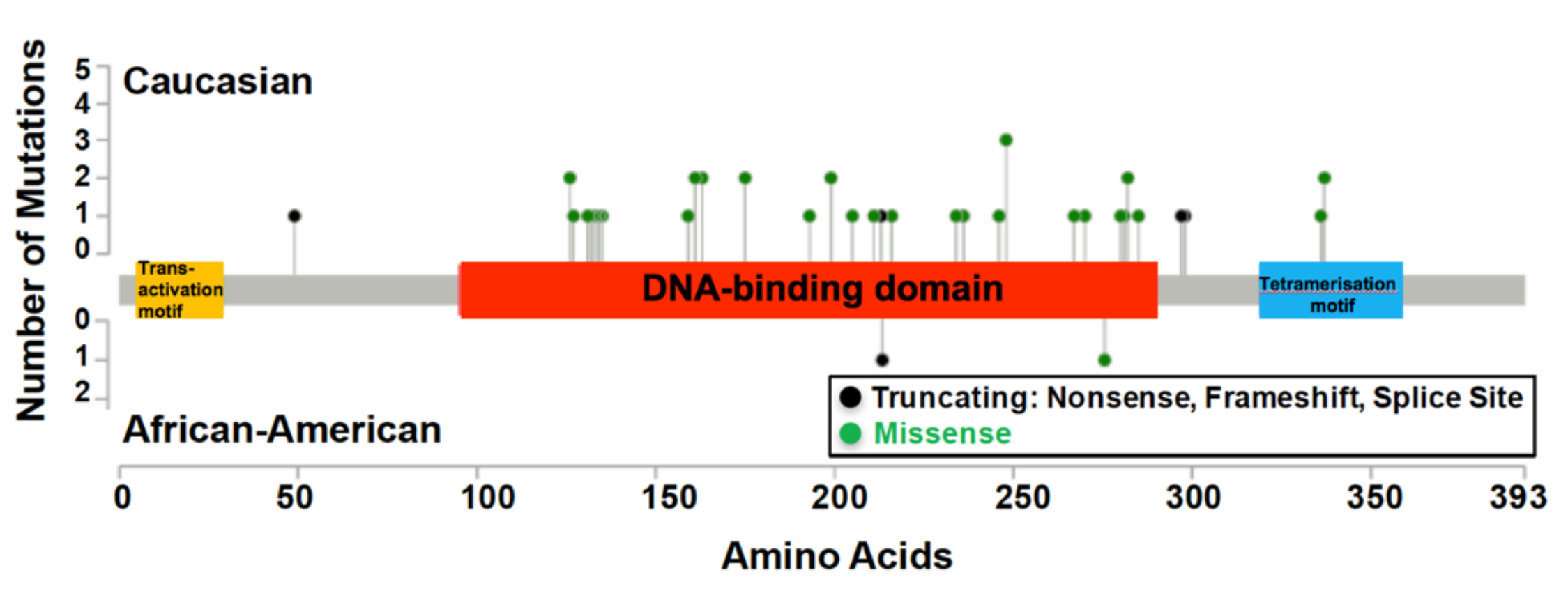

Supplement: S2 Fig — cBioPortal Mutation Mapper tool as described by Gao et al. Sci. Signal. 2013 & Cerami et al. Cancer Discov. 2012 was applied to generate the mutation profile across the TP53 domains with top representing mutation profile among Caucasian, and bottom representing African American respectively. (TIF) [file pgen.1007087.s002.tif]

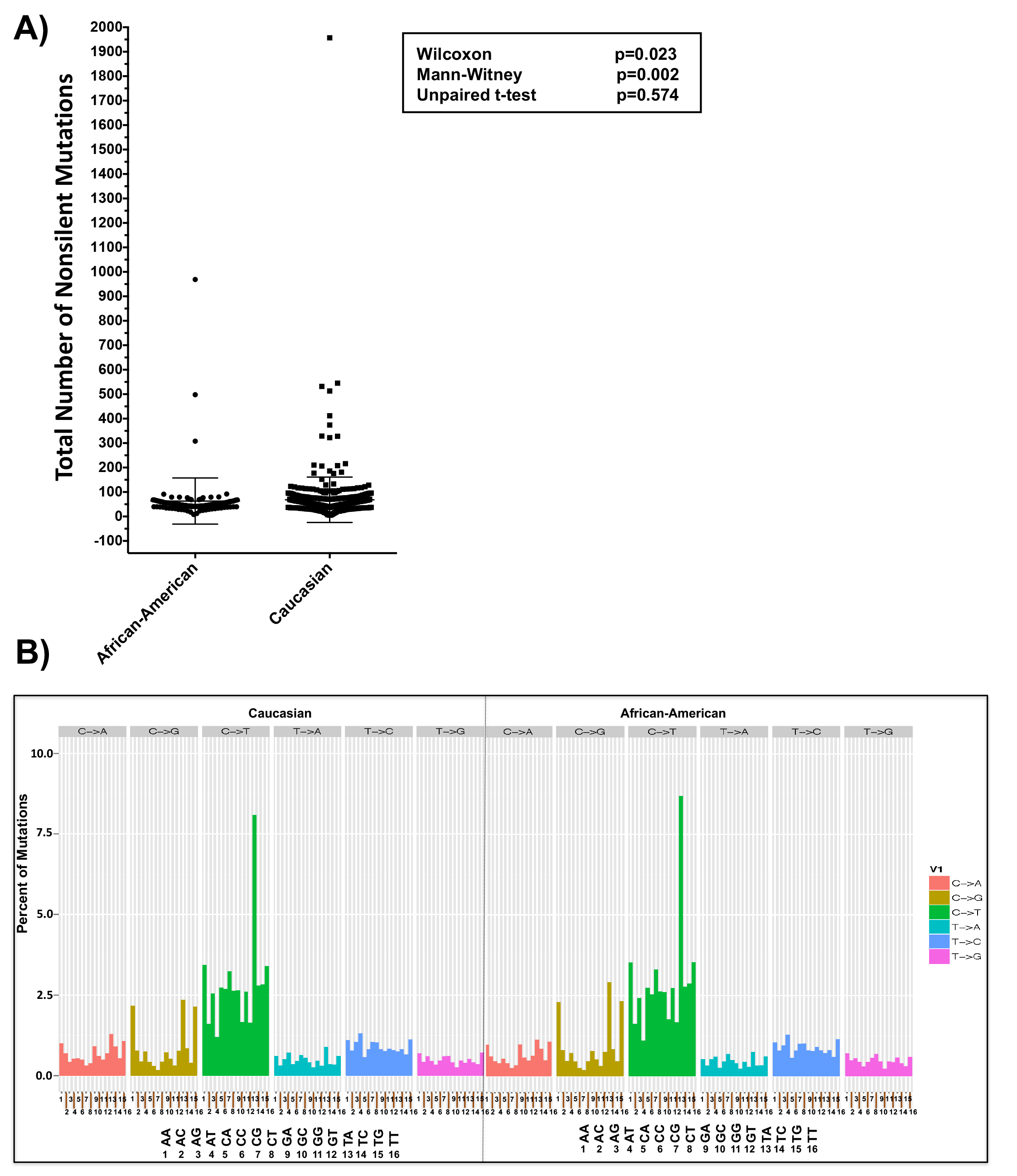

Supplement: S3 Fig — (A) Comparison of nonsilent mutation frequency between ancestry and self-reporting using Wilcoxon, Mann-Whitney, and Unpaired t-test to determine statistical significance. (B) Mutation signature associated with African and European ancestry. (TIF) [file pgen.1007087.s003.tif]

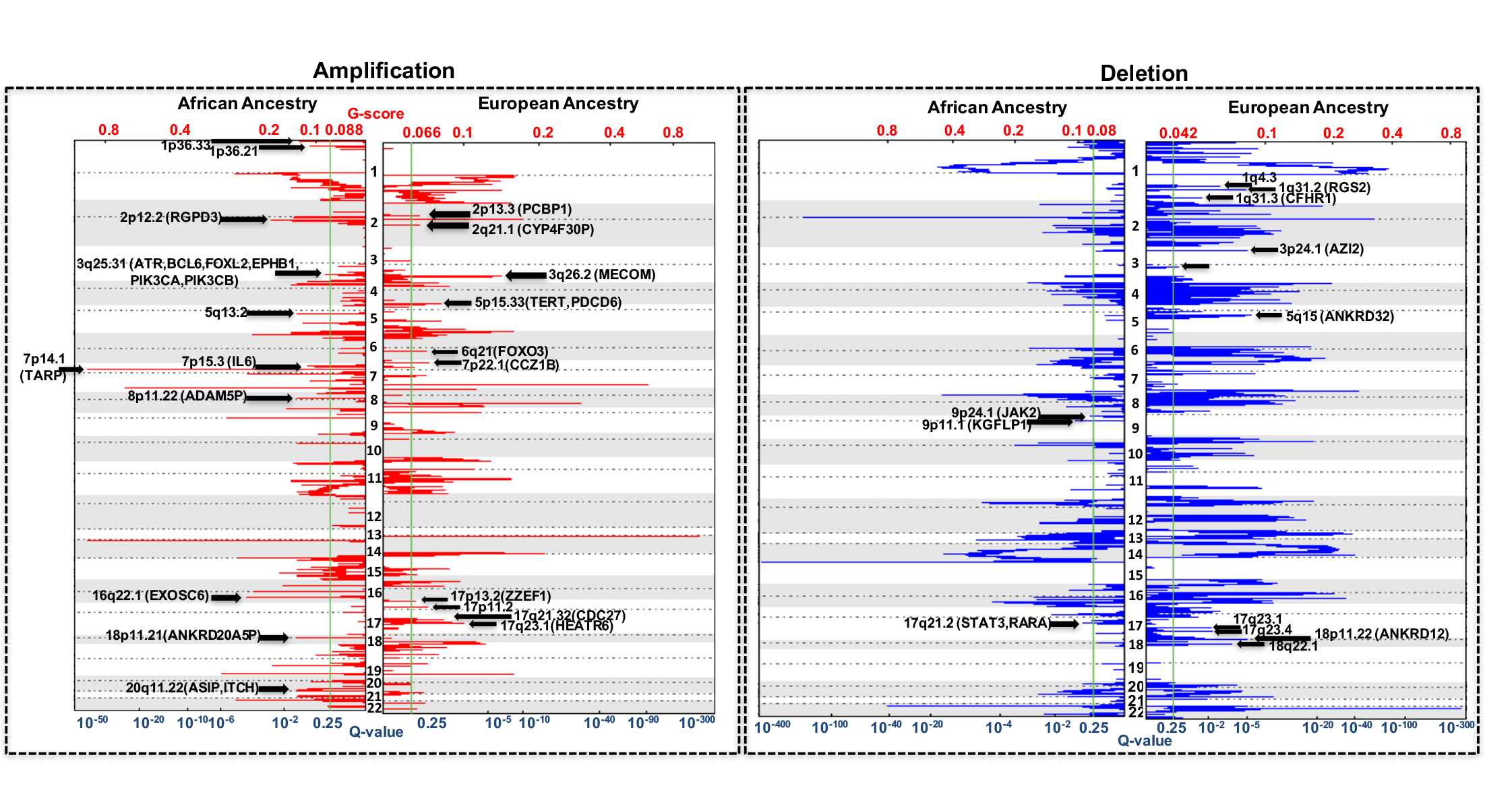

Supplement: S4 Fig — Differentially altered events indicated by arrows. The red graph indicate copy number gains and blue is deletions for each stratified group. (TIF) [file pgen.1007087.s004.tif]

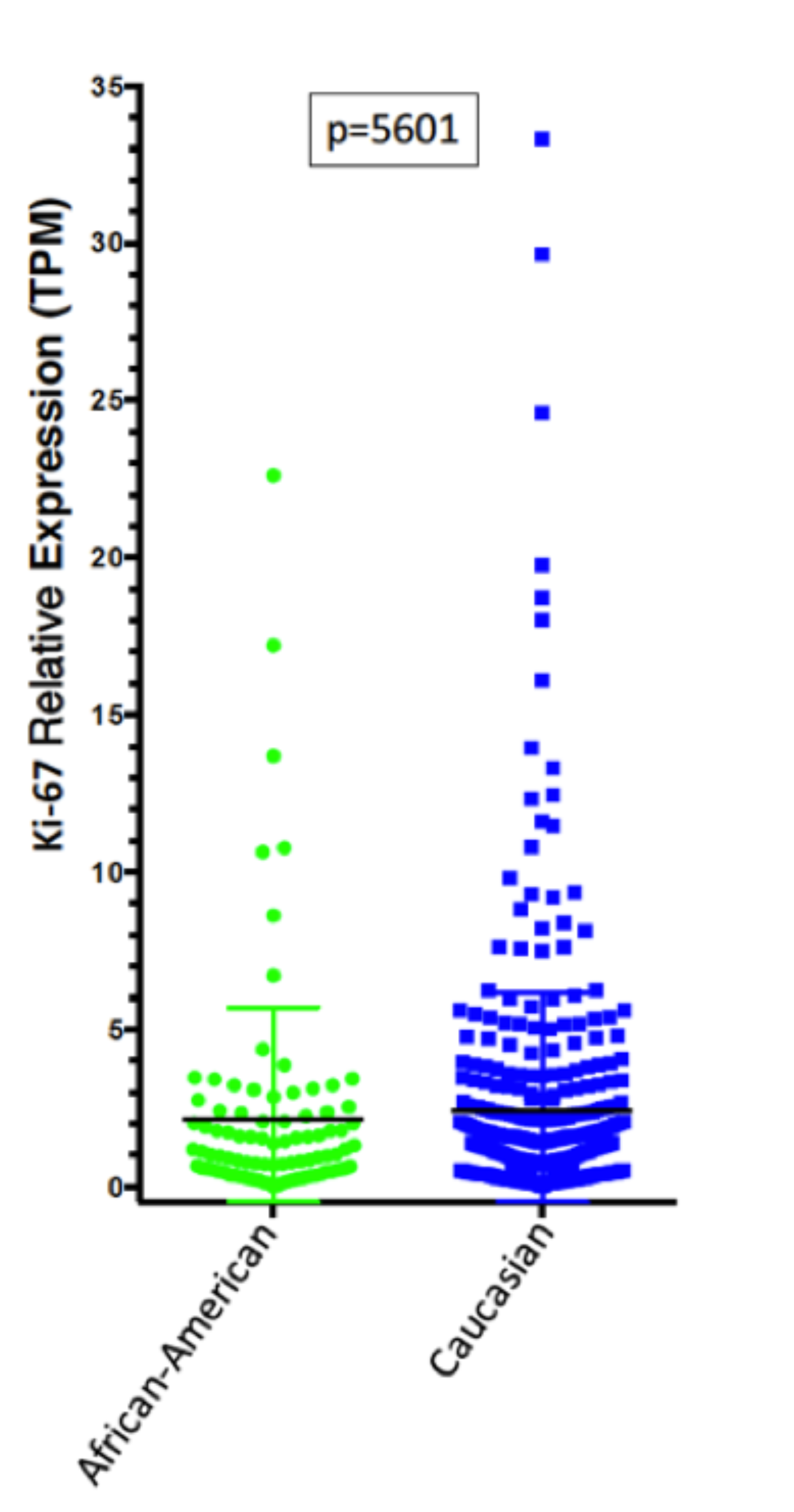

Supplement: S5 Fig — Ki67 expression profile in TPM across patients with MM. (TIF) [file pgen.1007087.s005.tif]
