# Supplementary material for: Comprehensive molecular profiling of 718 Multiple Myelomas reveals significant differences in mutation frequencies between African and European descent cases
Source: PLoS Genet. 2017 Nov 22;13(11):e1007087. doi: 10.1371/journal.pgen.1007087 (PMC5699827; doi:10.1371/journal.pgen.1007087)
Supplement: S3 Table — (PDF) [file pgen.1007087.s008.pdf]

S3 Table. Clinical Outline, Treatment summary, and karytype summary

Demographics	African-American	Caucasian	p-value (Fisher's)
**GENDER**			
**Male**	59.8% (76/127)	62.4% (369/591)	0.585
**Female**	40.1% (51/127)	37.5% (222/591)	0.585
**AGE(years)**			
**25-39**	3.1% (4/127)	1.7% (10/591)	0.282
**40-49**	11% (14/127)	4.6% (27/591)	0.004
**50-59**	21.3% (27/127)	24.5% (145/591)	0.433
**60-69**	38.6% (49/127)	37.9% (224/591)	0.886
**70-79**	14.2% (118/127)	22.3% (132/591)	0.040
**80-89**	11% (14/127)	8.1% (48/591)	0.291
**90-99**	0.8% (1/127)	0.8% (5/591)	0.948
**Treatment**			
**IMIDs**	6.3% (8/127)	5.4% (32/591)	0.694
**Carfilzomib**	1.6% (2/127)	1.7% (10/591)	0.926
**Bortezomib**	25.2% (32/127)	28.4% (168/591)	0.462
**Bortezomib/IMIDs**	57.5% (73/127)	56.5% (334/591)	0.842
**Carfilzomib/IMIDs**	5.5% (7/127)	4.9% (29/591)	0.777
**Carfilzomib/Bortezomib**	0.7% (1/127)	0.5% (3/591)	0.701
**Carfilzomib/IMIDs/Bortezomib**	3.1% (4/127)	2.5% (15/591)	0.697
**Karyotpye**			
**Hyperdiploid**	53.5% (68/127)	55.8% (303/591)	0.642
**Nonhyperdiplid**	46.4% (59/127)	48.7% (288/591)	0.642
