# Supplementary material for: Comprehensive molecular profiling of 718 Multiple Myelomas reveals significant differences in mutation frequencies between African and European descent cases
Source: PLoS Genet. 2017 Nov 22;13(11):e1007087. doi: 10.1371/journal.pgen.1007087 (PMC5699827; doi:10.1371/journal.pgen.1007087)
Supplement: S4 Table — (PDF) [file pgen.1007087.s009.pdf]

S4 Table. Summary of Significantly Mutated Genes

GeneName	African-American (Percent Mutation; n=127)	Caucasian (Percent Mutation n=591)	p-value (Fisher's)
KRAS	26.8%	23.7%	0.463
NRAS	22.8%	21.3%	0.707
FAM46C	12.6%	8.3%	0.093
RYR1*	9.4%	4.9%	0.045
DIS3	7.9%	10.0%	0.465
RPL10*	4.7%	1.0%	0.003
PTCHD3*	4.7%	1.0%	0.003
BCL7A*	3.9%	0.8%	0.007
SPEF2*	3.9%	0.8%	0.001
SP140	3.9%	2.5%	0.610
BRAF	3.9%	8.3%	0.092
MAX	3.9%	2.2%	0.319
TRAF3	3.9%	6.9%	0.430
LRP1B	3.9%	6.6%	0.257
PRKD2	3.9%	2.4%	0.319
MYH13*	3.9%	0.8%	0.007
ABI3BP*	3.9%	1.0%	0.015
BRWD3*	3.9%	0.8%	0.007
GRM7*	3.9%	1.0%	0.015
AUTS2*	3.9%	1.2%	0.028
PARP4*	3.9%	1.0%	0.015
PLD1*	3.1%	0.3%	0.002
ANKRD26*	3.1%	0.2%	0.0002
SETD2	3.1%	2.5%	0.697
SAMHD1	2.4%	1.9%	0.608
CSMD3	3.1%	6.4%	0.153
DDX17*	3.1%	0.7%	0.016
STXBP4*	3.1%	0.0%	0.00001
RB1	2.4%	1.0%	0.152
CYLD	1.6%	2.5%	0.518
TP53*	1.6%	6.3%	0.035
HIST1H4B	1.6%	0.3%	0.090
TBC1D29	0.8%	1.7%	0.452
ZNF292	0.8%	2.9%	0.172
PABPC1	0.8%	2.7%	0.226
RPL5	0.8%	1.4%	0.522
FAM111B	0.0%	0.2%	0.643
FBXO4	0.0%	0.5%	0.422
TRIAP1	0.0%	0.2%	0.643
TGDS	0.0%	1.5%	0.162
KDM3B	0.0%	2.5%	0.070
HIST1H2BH	0.0%	0.8%	0.299
WDR45	0.0%	1.0%	0.255
IRF4*	0.0%	3.2%	0.041

*** = p<0.05**
